# Effectiveness of repeated ultrasound-guided suprascapular nerve blocks in hemiplegic shoulder pain: A randomized controlled trial

**DOI:** 10.1097/MD.0000000000047547

**Published:** 2026-02-13

**Authors:** Alper T. Dogan, Yasemin Pekin Dogan, Ebru Aytekin, Selman Sogut, Zafer Gokkaya, Murat Tumer, Omur Ercelen, Yavuz Gurkan

**Affiliations:** a Department of Anesthesia and Intensive Care, Amerikan Hospital, Istanbul, Turkey; b Department of Physical Theraphy and Rehabilitation, Istanbul Education and Research Hospital, Istanbul, Turkey; c Department of Anesthesia and Intensive Care, Koc University Hospital, Istanbul, Turkey.

**Keywords:** hemiplegic shoulder pain, range of motion, suprascapular nerve block, ultrasound

## Abstract

**Background:**

Hemiplegic shoulder pain (HSP) is a common complication after stroke, significantly impairing rehabilitation. While suprascapular nerve block (SSNB) has shown promise in pain management, its long-term efficacy, especially with repeated applications, remains unclear.

**Objective:**

This study aimed to evaluate the effectiveness of ultrasound-guided repeated SSNB in reducing pain, improving range of motion (ROM), and enhancing motor function in patients with HSP.

**Methods:**

This double-blind, randomized controlled trial included 42 patients with HSP, allocated to either the SSNB group or the control group in a 1:1 ratio. The SSNB group received ultrasound-guided injections of levobupivacaine and triamcinolone at baseline, 3 weeks, and 6 weeks, while the control group received sham injections. All participants followed a standardized physiotherapy program. Pain was assessed using the visual analog scale, ROM with a goniometer, spasticity with the Ashworth scale, and motor recovery with the Brunnstrom scale. Assessments were conducted at baseline, 1, 4, and 7 weeks.

**Results:**

Thirty-nine patients completed the study. The SSNB group showed significant reductions in visual analog scale scores at 1, 4, and 7 weeks compared to the control group (*P* < .05). ROM for abduction and flexion significantly improved in the SSNB group at 7 weeks (*P* < .05). However, there were no significant differences between groups in Ashworth or Brunnstrom scores at any time point.

**Conclusion:**

Repeated SSNB effectively reduces pain and improves ROM in patients with HSP when combined with physiotherapy. However, it does not significantly impact spasticity or motor function. Further research with longer follow-ups and active comparators is needed to determine its long-term benefits.

**Key points**

– Question: Can repeated ultrasound-guided suprascapular nerve block (SSNB) reduce hemiplegic shoulder pain and improve range of motion (ROM) in stroke patients?
– Findings: Repeated SSNB significantly reduced pain and improved shoulder ROM, with no effect on spasticity or motor function.
– Meaning: Repeated SSNB effectively relieves pain and improves ROM in HSP, though it does not enhance motor recovery or reduce spasticity.

## 1. Introduction

Hemiplegic shoulder pain (HSP) is a common complication after a stroke, with an incidence ranging from 30% to 65%.^[1,2]^ It can arise from various biomechanical changes, including subluxation, adhesive capsulitis, heterotopic ossification, spasticity, and central or neuropathic pain mechanisms.^[3,4]^ Persistent HSP not only causes significant discomfort but also restricts range of motion (ROM), ultimately hindering rehabilitation efforts. The primary goals of HSP treatment are to alleviate pain, improve ROM, and enhance motor function while addressing spasticity.

A variety of treatments, including physical therapy modalities, analgesics, nonsteroidal anti-inflammatory drugs, and intraarticular injections, have been shown to be beneficial in many cases.^[5]^ However, no single treatment has proven significantly superior to the others.

Although earlier studies found that suprascapular nerve block (SSNB) was as effective as other treatments for HSP, it was not identified as superior.^[6–9]^ More recent findings, however, suggest that SSNB provides greater pain relief than placebo in reducing HSP intensity.^[10]^ Most prior studies on SSNB have focused on single-dose interventions, which have demonstrated pain relief. Repeated nerve blocks have been shown to be effective and safe in some pain syndromes without causing permanent nerve injury,^[11–13]^ but studies specifically investigating repetitive SSNB for HSP are limited and insufficient.

This study aimed to evaluate the effectiveness of ultrasound-guided repetitive suprascapular nerve blocks in patients with hemiplegic shoulder pain.

## 2. Methods

This study was conducted as a parallel design, double-blind, randomized controlled trial. Participants were allocated in a 1:1 ratio to either the suprascapular block group or the control group.

### 2.1. Participants

The study was approved by the Istanbul Education and Research Hospital Ethics Committee (16/03/2012–91), written informed consent was obtained from all subjects and conducted in an in-patient setting in the Physical Therapy and Rehabilitation Clinic of a tertiary care hospital in Turkey. Participants aged 18 to 80 years with hemiplegic shoulder pain following a stroke were eligible for inclusion in the study. Exclusion criteria included neuropathic pain, the presence of pressure ulcers, advanced degenerative changes in the glenohumeral joint as identified by anteroposterior shoulder radiography, and pain attributed to changes in the shoulder capsule or soft tissues, as supported by musculoskeletal examination and ultrasonography. Patients with infections (e.g., urinary or respiratory), cognitive dysfunction, or communication difficulties were also excluded. To assess cognitive dysfunction, the Mini-Mental State Examination was administered to all participants. Patients scoring <24 points on the test were excluded from the study.

### 2.2. Interventions

**Figure 1.**
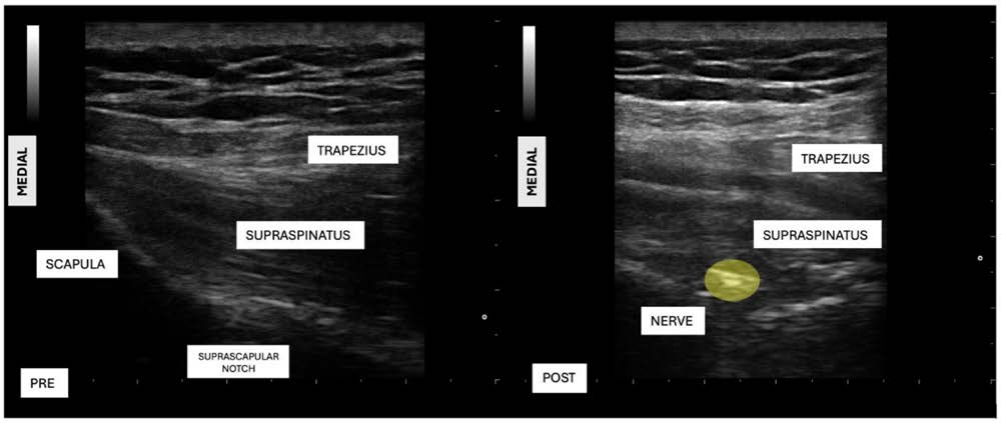
Suprascapular nerve block ultrasound images. Pre = before the procedure, Post = after local anesthetic or saline application, Nerve = suprascapular nerve, trapezius, and supraspinatus muscle.

In suprascapular block group, patients received a suprascapular nerve block while seated. A linear ultrasound probe (E-saote My Lab 5) was positioned transversely over the scapular spinous process. The suprascapular nerve was identified beneath the trapezius and supraspinatus muscles at the scapular notch. After subcutaneous administration of 2 mL of 2% lidocaine, an 80 mm block needle was advanced in an in-plane approach to the nerve, and 5 mL of 0.5% levobupivacaine combined with 40 mg triamcinolone acetonide was injected Fig. 1).

In the control group, patients underwent a sham procedure. While seated, the ultrasound probe was positioned transversely over the scapular spinous process, and 2 mL of 2% lidocaine was injected subcutaneously. An 80 mm peripheral nerve needle was advanced subcutaneously, and 5 mL of saline was injected into the muscle.

Interventions were performed 3 times for each patient: after the baseline examination, at 3 weeks, and at 6 weeks. Throughout the 7-week study, all patients followed a standardized range of motion exercise program.

### 2.3. Outcomes

#### 2.3.1. Primary outcome

The primary outcome was pain, evaluated using the visual analog scale (VAS). The VAS is a validated tool for measuring pain intensity, where participants mark their pain level on a 10 cm line, ranging from 0 (no pain) to 10 (worst possible pain).

#### 2.3.2. Secondary outcome

The secondary outcome was shoulder range of motion (ROM) for abduction and flexion, measured in degrees using a goniometer. ROM assessments were conducted by trained clinicians under standardized conditions.

#### 2.3.3. Exploratory outcomes

Exploratory outcomes included spasticity levels and motor recovery. Spasticity levels were assessed using the Ashworth scale, a clinical measure that evaluates muscle tone on a scale from 0 (no increase in muscle tone) to 4 (rigid in flexion or extension).^[14]^ Motor recovery was evaluated using the Brunnstrom scale, which classifies motor function recovery in hemiplegic patients across 6 stages for the upper extremity, lower extremity, and hand.^[15]^

All outcomes were assessed at baseline, and 1-, 4-, and 7-weeks post-baseline by clinicians blinded to group allocation.

### 2.4. Sample size calculation

The sample size was calculated based on a previous study, which reported a mean VAS score of 6 ± 2.2 for hemiplegic shoulder pain patients after 4 weeks of physical therapy.^[16]^ To detect a clinically significant reduction of 2 points in VAS scores with an alpha value of 0.05 and a beta value of 0.2, it was determined that 42 patients (21 in each group) were required for the study.

### 2.5. Randomization and blinding

Simple randomization was used to allocate participants to the suprascapular block group or the control group. The random allocation sequence was generated using a computer-based random number generator. No blocking or other restrictions were applied.

Blinding was maintained for participants, care providers, and outcome assessors. Outcome assessments were conducted by clinicians who were unaware of the group assignments. Only the anesthesiologists who were performing the blocks were not blinded due to the nature of the procedure, but they were not participated in any other aspect of the care or outcome assessment.

### 2.6. Statistical analysis

The primary outcome, pain levels measured using VAS scores were analyzed at 1, 4, and 7 weeks using the Mann–Whitney *U* test due to the nonparametric nature of the data. The secondary outcome, range of motion was analyzed using independent samples *t* tests at each follow-up time point. Exploratory outcomes were analyzed using the Mann–Whitney *U* test due to the nonparametric nature of the data. Adjusted *P*-values for all outcomes were calculated using Holm method to account for multiple comparisons, considering a total of 28 tests. Statistical significance was determined based on a 2-tailed adjusted *P*-value of less than .05. All analyses were performed using SPSS v20 software and results were presented as medians with interquartile ranges (M[IQR]) or means with standard deviations (M ± SD), as appropriate by their distribution.

### 2.7. Funding and conflict of interest

None of the authors have conflict of interest and there is no funding for the study.

## 3. Results

**Figure 2.**
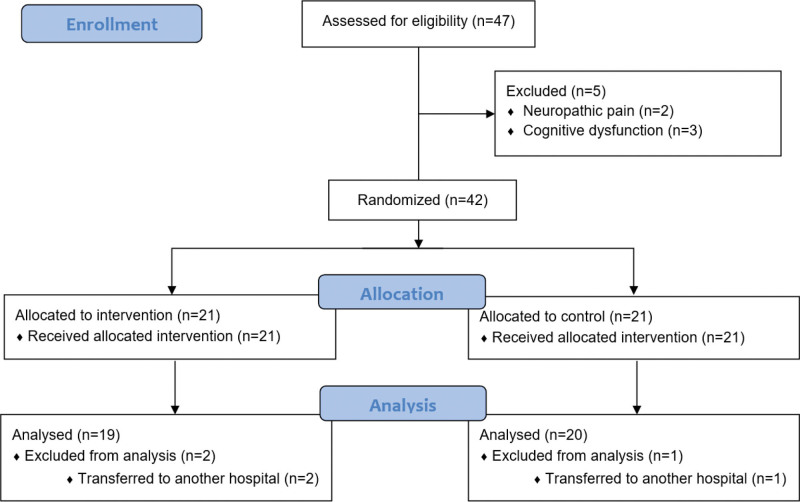
CONSORT 2010 flow diagram.

A total of 42 patients were enrolled in the study, with 19 in the suprascapular block group and 20 in the control group completing the trial. Two patients from the suprascapular block group and 1 patient from the control group were lost to follow-up due to transfer to other hospitals (Fig. 2). The mean age of patients in the suprascapular block group was 65.2 ± 8.35 years, compared to 69.4 ± 8.72 years in the control group (*t*(37) = −1.55, *P* = .130). Nineteen patients were male and twenty were female. The distribution of gender between the groups was not statistically significant (*χ*^2^(1, N = 39) = 0.027, *P* = .870).

Baseline assessments for range of motion, VAS scores, Ashworth scores, and Brunnstrom scale scores showed no statistically significant differences between the SSNB and the control groups. The detailed results, including adjusted *P*-values, are presented in Table 1.

**Table 1:** Baseline measurements of the patients. Results are presented as mean ± standard deviation or median (interquartile range), as appropriate by their distribution.

Outcome	Suprascapular block	Control	Adjusted *P*-value
Range of motion			
Abduction (°)	94.5 ± 21.0	97.5 ± 16.5	1.000
Flexion (°)	100.5 ± 25.9	103.0 ± 19.2	1.000
VAS scores	8.0 (1.5)	7.0 (1.0)	1.000
Ashworth scores	1.0 (1.0)	2.0 (1.0)	1.000
Brunnstrom scale			
Upper extremity	3.0 (1.5)	3.0 (1.0)	1.000
Lower extremity	4.0 (2.0)	3.0 (1.0)	.645
Hand	2.0 (1.0)	2.0 (1.0)	.522

VAS = visual analog scale.

### 3.1. Visual analog scale scores at follow-up

**Figure 3.**
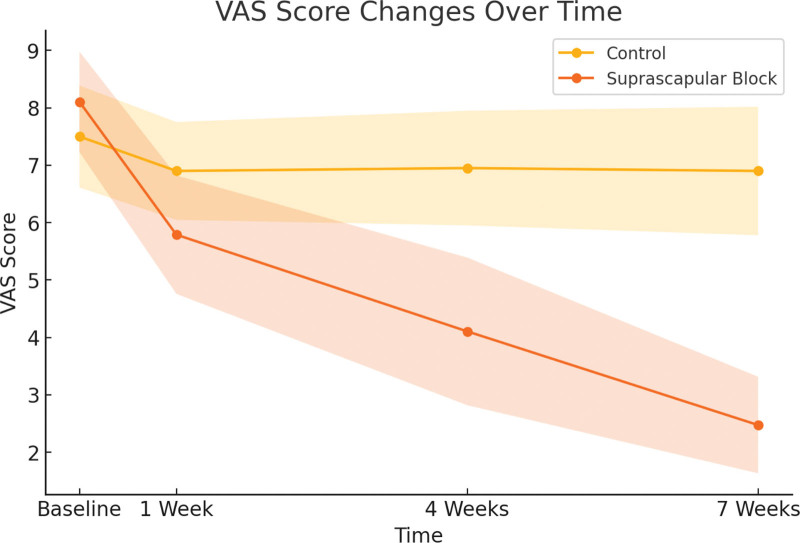
VAS score changes over time. VAS = visual analog scale.

VAS scores were assessed at 1, 4, and 7 weeks. Statistically significant differences in favor of SSNB were observed between the SSNB and control groups at all follow-up points. Detailed scores and *P*-values are presented in Figure 3 and Table 2.

**Table 2:** VAS scores at 1, 4, and 7 weeks. Results are presented as median (interquartile range).

Time point	Suprascapular block (median, IQR)	Control (median, IQR)	Adjusted *P*-value
1 Week	6.0 (1.5)	7.0 (1.0)	.048
4 Weeks	4.0 (2.0)	7.0 (2.0)	.028
7 Weeks	2.0 (1.0)	7.0 (2.0)	.028

VAS = visual analog scale, IQR = interquartile range.

### 3.2. Range of motion at follow-up

ROM for shoulder abduction and flexion was assessed at 1, 4, and 7 weeks. Statistically significant differences favoring the SSNB group were observed at 7 weeks for both abduction and flexion. Detailed scores and *P*-values are presented in Table 3.

**Table 3:** Range of motion at 1, 4, and 7 weeks. Results are presented as median (interquartile range).

Time point	Measurement	Suprascapular block (mean, SD)	Control (mean, SD)	Adjusted *P*-value
1 Week	Abduction	101.6° (18.0)	99.0° (16.5)	1.000
	Flexion	113.2° (20.8)	106.0° (16.0)	1.000
4 Weeks	Abduction	112.1° (18.7)	103.0° (15.9)	1.000
	Flexion	121.6° (20.1)	109.0° (17.1)	.585
7 Weeks	Abduction	123.2° (15.7)	106.5° (13.9)	.03
	Flexion	137.9° (18.1)	109.5° (16.1)	.028

### 3.3. Ashworth scores at follow-up

At all follow-up time points, the median Ashworth score in the suprascapular block group was 1.0 (1.0), while the control group had a median score of 2.0 (1.0). Despite consistent differences in median scores favoring the suprascapular block group, the Mann–Whitney *U* test did not identify any statistically significant differences between the groups at 1 week (*U* = 105.0, *P* = .181), 4 weeks (*U* = 105.0, *P* = .181), or 7 weeks (*U* = 105.0, *P* = .181).

### 3.4. Brunnstrom scale scores at follow-up

Brunnstrom scale scores were assessed at 1, 4, and 7 weeks for the upper extremity, lower extremity, and hand. No statistically significant differences were observed at any time point. Detailed scores and *P*-values are provided in Table 4.

**Table 4:** Brunnstrom scale scores at 1, 4, and 7 weeks.

Time point	Measurement	Supracscapular block (median, IQR)	Control (median, IQR)	Adjusted *P*-value
1 Week	Upper extremity	3.0 (1.0)	3.0 (1.0)	1
	Lower extremity	4.0 (2.0)	4.0 (1.0)	1
	Hand	2.0 (1.0)	2.0 (1.0)	.236
4 Weeks	Upper extremity	3.0 (1.0)	3.0 (0.0)	1
	Lower extremity	4.0 (1.0)	4.0 (0.25)	.931
	Hand	2.0 (1.0)	2.0 (1.0)	.169
7 Weeks	Upper extremity	3.0 (1.0)	3.0 (0.0)	1
	Lower extremity	4.0 (1.0)	4.0 (0.0)	.378
	Hand	3.0 (1.0)	2.0 (1.0)	.101

IQR = interquartile range.

## 4. Discussion

This study aimed to evaluate the effectiveness of repeated ultrasound-guided suprascapular nerve block applications in improving range of motion, reducing pain as measured by VAS scores, alleviating spasticity assessed by Ashworth scores, and enhancing motor function as assessed by Brunnstrom scale scores.

The repeated SSNB applications significantly reduced pain at 1, 4, and 7 weeks compared to the control group, aligning with findings from existing literature.^[17,18]^ The lowest VAS scores in the SSNB group were observed at week 7, consistent with previous studies.^[19]^

Unlike studies that demonstrated pain reduction with single-dose SSNB but found no significant effects on secondary outcomes,^[8,20]^ results of this study showed that repeated SSNB combined with physiotherapy significantly improved ROM in both abduction and flexion by week 7. These results differ from studies comparing single-dose SSNB to Botulinum toxin A, which reported similar effects on pain and ROM up to week 2, but diminished benefits by weeks 4 and 6.^[21,22]^ The superior results observed at week 7 are attributed to the repeated SSNB applications facilitating adherence to the range of motion exercise program. This finding aligns with studies showing improved outcomes in chronic shoulder pain and frozen shoulder patients treated with continuous SSNB and physiotherapy over 12 weeks, as measured by the shoulder pain and disability index.^[23,24]^

In contrast to studies demonstrating that ultrasound-guided Botulinum toxin A injections significantly reduce spasticity and improve motor function,^[25]^ no improvements in spasticity or motor recovery were observed with SSNB, similar to findings reported by Aydin et al.^[26]^ The lack of improvement in spasticity may be explained by the mechanism of SSNB, which facilitates physiotherapy and improves ROM by reducing pain but does not directly prevent spasticity as Botulinum toxin A does.

### 4.1. Limitations

This study has several limitations. The small sample size and conducting the study in a single tertiary hospital may limit the generalizability of the results. The follow-up period was restricted to 7 weeks, preventing the assessment of long-term effects. While shoulder range of motion was measured, functional outcomes such as daily activity improvements were not evaluated. Additionally, the anesthesiologists performing the interventions were not blinded, introducing potential bias. Lastly, the control group received a sham injection rather than an active comparator, limiting direct comparisons with other treatments. Future studies with larger cohorts, longer follow-ups, and active comparators are needed to strengthen these findings.

## 5. Conclusion

SSNB is a safe, effective, and practical treatment option for patients with hemiplegic shoulder pain. Repeated SSNB applications provide better pain control and result in improved ROM outcomes, compared to placebo.

## Author contributions

**Conceptualization:** Alper T. Dogan, Yasemin Pekin Dogan, Ebru Aytekin.

**Data curation:** Alper T. Dogan, Yasemin Pekin Dogan, Selman Sogut, Zafer Gokkaya.

**Formal analysis:** Alper T. Dogan, Yasemin Pekin Dogan, Selman Sogut, Murat Tumer, Omur Ercelen, Yavuz Gurkan.

**Investigation:** Alper T. Dogan, Yasemin Pekin Dogan.

**Methodology:** Alper T. Dogan, Yasemin Pekin Dogan, Ebru Aytekin.

**Supervision:** Alper T. Dogan, Yasemin Pekin Dogan.

**Validation:** Alper T. Dogan.

**Visualization:** Alper T. Dogan.

**Writing—original draft:** Alper T. Dogan, Selman Sogut, Zafer Gokkaya.

**Writing—review & editing:** Alper T. Dogan, Yasemin Pekin Dogan, Ebru Aytekin, Selman Sogut, Zafer Gokkaya, Murat Tumer, Omur Ercelen, Yavuz Gurkan.
